# Supplementary material for: Evidence on the formation of dimers of polycyclic aromatic hydrocarbons in a laminar diffusion flame
Source: Commun Chem. 2020 Aug 11;3:112. doi: 10.1038/s42004-020-00357-2 (PMC9814144; doi:10.1038/s42004-020-00357-2)
Supplement: Supplementary file 1 — Supplementary Information [file 42004_2020_357_MOESM1_ESM.pdf]

Supplementary Information

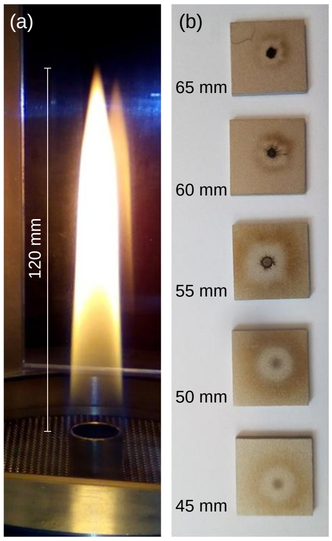

Supplementary Figure 1. Pictures of (a) investigated laminar diffusion methane flame, and (b) samples extracted from the flame axis showing the transition from condensable gas to soot particles. 45 and 50 mm HAB: gas-phase precursors region, close to the UV-LIF maximum and between the maxima of UV-LIF and Vis-LIF signals, respectively. 55 mm HAB: transition region, beginning of the detection of soot particles by LII. 60 and 65 mm HAB: soot region at the beginning of the LII signal and before the LII signal maximum at 80 mm HAB, respectively.

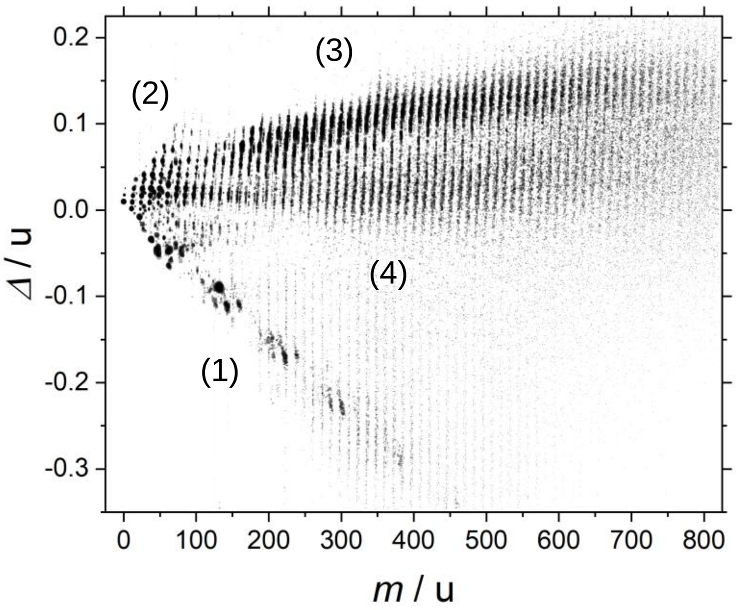

Supplementary Figure 2. Mass defect plot showing the ensemble of detected peaks having SNR > 3. The size of the datapoints is proportional to the logarithm of the peak intensity. The low mass defect series (1), also very prominent in the blank, is easily assigned to Ti oxides from the deposition substrates. The low *m*/*z* signals (2) are mostly assigned to the blank and fragment ions. The high *m*/*z* and large mass defect signals (3) are typical of soot and contain many identified hydrocarbons $C_mH_n^+$. Finally, the high *m*/*z* and low mass defect signals (4), also typical of soot, are assigned to oxygen-containing hydrocarbons $C_mH_nO_p^+$ and/or carbon cluster ions.

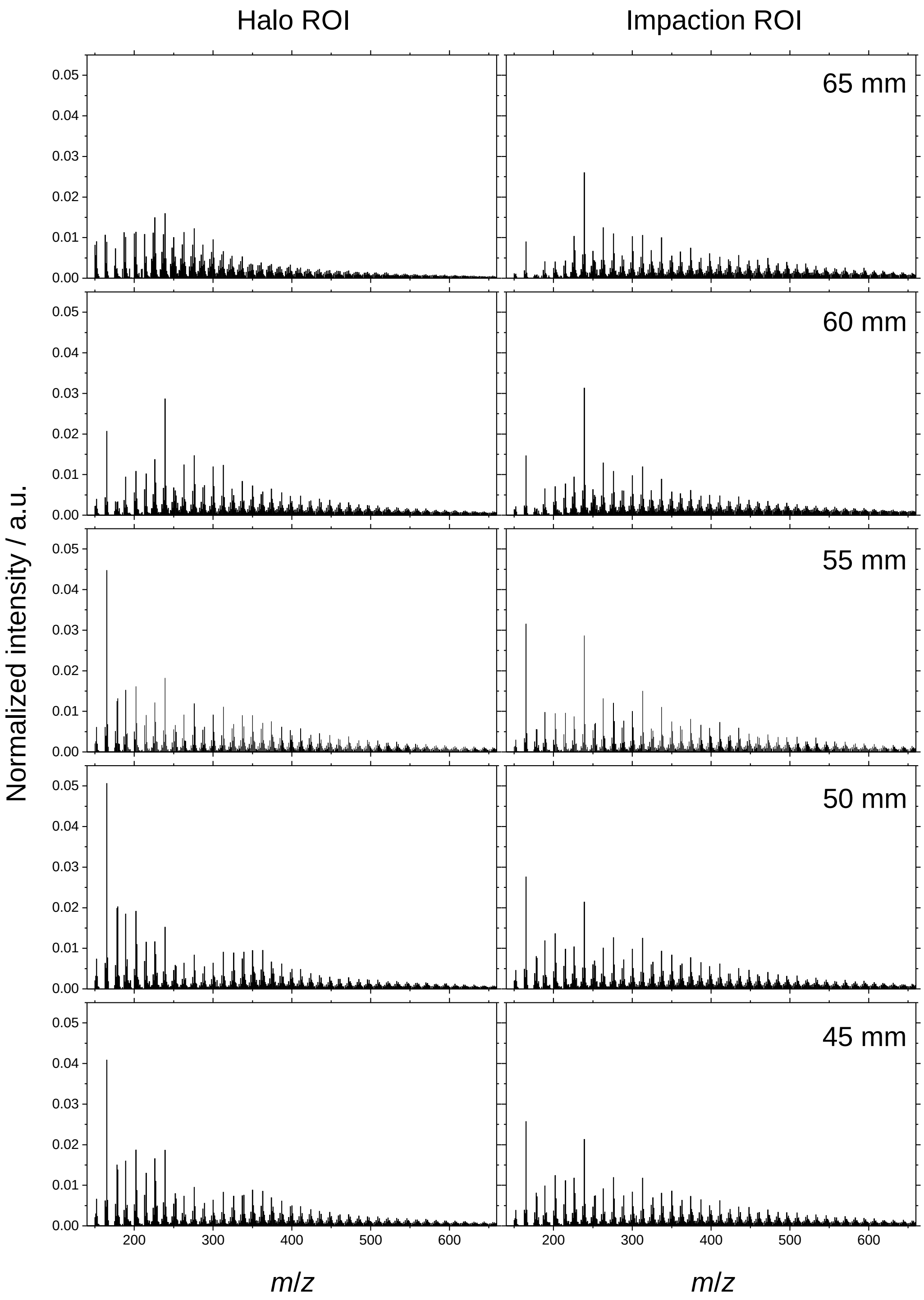

Supplementary Figure 3. Normalized mass spectra after blank and fragment ions removal. Left column: halo ROI, right column: impaction ROI. Each row corresponds to one different sampling HAB.

m/z	Formula	*m/z*	Formula	*m/z*	Formula	*m/z*	Formula	*m/z*	Formula
150.04(2)	$C_{12}H_6^+$	273.05(3)	$[^{13}C]C_{18}H_8O_3^+$	381.13(4)	?	496.11(5)	$C_{40}H_{16}^+$	619.13(6)	$[^{13}C]C_{50}H_{18}^+$
151.05(2)	$[^{13}C]C_{12}H_6^+$	274.07(3)	$C_{18}H_{10}O_3^+$	382.01(4)	?	497.11(5)	$[^{13}C]C_{40}H_{16}^+$	620.14(6)	$C_{50}H_{20}^+$
152.06(2)	$C_{12}H_8^+$	275.07(3)	$[^{13}C]C_{18}H_{10}O_3^+$	383.03(4)	?	498.13(5)	$C_{40}H_{18}^+$	621.13(6)	$[^{13}C]C_{50}H_{20}^+$
153.07(2)	$[^{13}C]C_{12}H_8^+$	276.09(3)	$C_{22}H_{12}^+$	384.05(4)	?	499.13(5)	$[^{13}C]C_{40}H_{18}^+$	622.15(6)	$C_{50}H_{22}^+$
154.08(2)	$C_{12}H_{10}^+$	277.10(3)	$[^{13}C]C_{22}H_{12}^+$	385.08(4)	$C_{27}H_{13}O_3^+$	500.13(5)	$C_{40}H_{20}^+$	623.15(6)	$[^{13}C]C_{50}H_{22}^+$
155.09(2)	?	278.10(3)	$C_{22}H_{14}^+$	386.09(4)	$[^{13}C]C_{27}H_{13}O_3^+$	501.01(5)	?	624.16(6)	?
163.02(2)	$C_{12}H_3O^+$	279.11(3)	$[^{13}C]C_{22}H_{14}^+$	387.11(4)	$C_{31}H_{15}^+$	501.15(5)	$[^{13}C]C_{40}H_{20}^+$	625.03(6)	?
163.05(2)	$C_{13}H_7^+$	280.12(3)	?	388.11(4)	$[^{13}C]C_{31}H_{15}^+$	502.15(5)	?	625.16(6)	?
164.03(2)	$[^{13}C]C_{12}H_3O^+$	280.99(3)	?	389.12(4)	$C_{31}H_{17}^+$	503.01(5)	?	627.04(6)	?
164.06(2)	$[^{13}C]C_{13}H_7^+$	281.1(3)	$C_{21}H_{13}O^+$	390.12(4)	$[^{13}C]C_{31}H_{17}^+$	505.03(5)	?	629.08(6)	?
165.07(2)	$C_{13}H_9^+$	282.01(3)	?	391.13(4)	$C_{31}H_{19}^+$	506.04(5)	?	630.07(6)	?
166.08(2)	$[^{13}C]C_{13}H_9^+$	283.01(3)	?	392.00(4)	?	507.06(5)	?	631.13(6)	$C_{51}H_{19}^+$
167.09(2)	?	284.03(3)	?	392.14(4)	$[^{13}C]C_{31}H_{19}^+$	508.10(5)	?	632.14(6)	$[^{13}C]C_{51}H_{19}^+$
175.01(2)	$[^{13}C]C_{13}H_2O^+$	285.01(3)	?	393.01(4)	?	509.13(5)	$C_{41}H_{17}^+$	633.15(6)	$C_{51}H_{21}^+$
176.03(2)	$C_{13}H_4O^+$	285.05(3)	$C_{19}H_9O_3^+$	393.13(4)	?	510.12(5)	$[^{13}C]C_{41}H_{17}^+$	634.14(6)	$[^{13}C]C_{51}H_{21}^+$
176.06(2)	$C_{14}H_8^+$	286.06(3)	$[^{13}C]C_{19}H_{10}O_3^+$	394.02(4)	?	511.14(5)	$C_{41}H_{19}^+$	635.16(6)	$C_{51}H_{23}^+$
177.04(2)	$[^{13}C]C_{13}H_4O^+$	287.08(3)	$C_{23}H_{11}^+$	395.02(4)	?	512.14(5)	$[^{13}C]C_{41}H_{19}^+$	636.02(6)	?
177.06(2)	$[^{13}C]C_{14}H_8^+$	288.09(3)	$[^{13}C]C_{23}H_{11}^+$	396.05(4)	$C_{24}H_{12}O_6^+$	513.14(5)	$C_{41}H_{20}^+$	636.14(6)	$[^{13}C]C_{51}H_{23}^+$
178.08(2)	$C_{14}H_{10}^+$	289.11(3)	$C_{23}H_{13}^+$	397.05(4)	$[^{13}C]C_{24}H_{12}O_6^+$	514.01(5)	?	637.16(6)	?
179.09(2)	$[^{13}C]C_{14}H_{10}^+$	290.11(3)	$[^{13}C]C_{23}H_{13}^+$	398.10(4)	$C_{32}H_{14}^+$	514.13(5)	$[^{13}C]C_{41}H_{21}^+$	638.05(6)	?
180.09(2)	?	291.11(3)	?	399.11(4)	$[^{13}C]C_{32}H_{14}^+$	516.04(5)	?	640.06(6)	?
181.07(2)	$C_{13}H_9O^+$	292.10(3)	$C_{22}H_{12}O^+$	400.12(4)	$C_{32}H_{16}^+$	517.03(5)	?	641.04(6)	?
182.07(2)	$[^{13}C]C_{13}H_9O^+$	293.10(3)	$[^{13}C]C_{22}H_{12}O^+$	401.12(4)	$[^{13}C]C_{32}H_{16}^+$	518.04(5)	?	642.10(6)	$C_{44}H_{18}O_6^+$
185.04(2)	?	294.00(3)	?	402.13(4)	$C_{32}H_{18}^+$	519.05(5)	?	643.11(6)	$[^{13}C]C_{44}H_{18}O_6^+$
187.01(2)	?	295.01(3)	?	403.00(4)	?	520.10(5)	$C_{42}H_{16}^+$	644.15(6)	$C_{52}H_{20}^+$
187.05(2)	$C_{15}H_7^+$	296.01(3)	?	403.14(4)	$[^{13}C]C_{32}H_{18}^+$	521.11(5)	$[^{13}C]C_{42}H_{16}^+$	645.15(6)	$[^{13}C]C_{52}H_{20}^+$
188.03(2)	?	297.03(3)	?	404.14(4)	?	522.13(5)	$C_{42}H_{18}^+$	646.15(6)	$C_{52}H_{22}^+$
188.05(2)	$[^{13}C]C_{15}H_7^+$	298.06(3)	$C_{20}H_{10}O_3^+$	405.01(4)	?	523.14(5)	$[^{13}C]C_{42}H_{18}^+$	647.14(6)	$[^{13}C]C_{52}H_{22}^+$
189.07(2)	$C_{15}H_9^+$	299.06(3)	$[^{13}C]C_{20}H_{10}O_3^+$	405.12(4)	?	524.13(5)	$C_{42}H_{20}^+$	648.16(6)	$C_{52}H_{24}^+$
190.08(2)	$[^{13}C]C_{15}H_9^+$	300.09(3)	$C_{24}H_{12}^+$	406.02(4)	?	525.14(5)	$[^{13}C]C_{42}H_{20}^+$	649.02(6)	?
191.09(2)	$C_{15}H_{11}^+$	301.10(3)	$[^{13}C]C_{24}H_{12}^+$	407.03(4)	?	526.14(5)	?	649.17(6)	$[^{13}C]C_{52}H_{24}^+$
192.10(2)	$[^{13}C]C_{15}H_{11}^+$	302.11(3)	$C_{24}H_{14}^+$	408.02(4)	?	527.01(5)	?	651.04(7)	?
193.10(2)	?	303.12(3)	$[^{13}C]C_{24}H_{14}^+$	409.07(4)	$C_{25}H_{13}O_6^+$	527.14(5)	?	653.05(7)	?
194.01(2)	$C_{16}H_2^+$	304.12(3)	?	410.08(4)	$[^{13}C]C_{25}H_{13}O_6^+$	528.02(5)	?	654.08(7)	?
194.08(2)	$C_{14}H_{10}O^+$	304.99(3)	?	411.11(4)	$C_{33}H_{15}^+$	529.02(5)	?	655.14(7)	$C_{53}H_{19}^+$
195.09(2)	$[^{13}C]C_{14}H_{10}O^+$	305.10(3)	$C_{23}H_{13}O^+$	412.12(4)	$[^{13}C]C_{33}H_{15}^+$	530.03(5)	?	656.14(7)	$[^{13}C]C_{53}H_{19}^+$
196.09(2)	?	306.10(3)	$[^{13}C]C_{23}H_{13}O^+$	413.12(4)	$C_{33}H_{17}^+$	531.05(5)	?	657.12(7)	$C_{53}H_{21}^+$
200.06(2)	$C_{16}H_8^+$	307.01(3)	?	414.13(4)	$[^{13}C]C_{33}H_{17}^+$	532.09(5)	?	658.14(7)	$[^{13}C]C_{53}H_{21}^+$
201.04(2)	?	308.03(3)	?	415.13(4)	$C_{33}H_{19}^+$	533.12(5)	$C_{43}H_{17}^+$	659.14(7)	$C_{53}H_{23}^+$
201.06(2)	$[^{13}C]C_{16}H_8^+$	309.02(3)	?	416.01(4)	?	534.13(5)	$[^{13}C]C_{43}H_{17}^+$	660.15(7)	$[^{13}C]C_{53}H_{23}^+$
202.08(2)	$C_{16}H_{10}^+$	310.05(3)	?	416.13(4)	$[^{13}C]C_{33}H_{19}^+$	535.14(5)	$C_{43}H_{19}^+$	661.17(7)	?
203.09(2)	$[^{13}C]C_{16}H_{10}^+$	311.08(3)	$C_{21}H_{11}O_3^+$	417.14(4)	?	536.14(5)	$[^{13}C]C_{43}H_{19}^+$	662.04(7)	?
204.09(2)	$C_{16}H_{12}^+$	312.08(3)	$[^{13}C]C_{21}H_{11}O_3^+$	418.01(4)	?	537.15(5)	$C_{43}H_{21}^+$	664.06(7)	?
205.07(2)	$C_{15}H_9O^+$	313.10(3)	$C_{25}H_{13}^+$	419.02(4)	?	538.01(5)	?	666.10(7)	$C_{46}H_{18}O_6^+$
205.10(2)	$[^{13}C]C_{16}H_{12}^+$	314.10(3)	$[^{13}C]C_{25}H_{13}^+$	420.03(4)	?	538.15(5)	$[^{13}C]C_{43}H_{21}^+$	667.11(7)	$[^{13}C]C_{46}H_{18}O_6^+$
206.01(2)	$C_{17}H_2^+$	315.12(3)	$C_{25}H_{15}^+$	421.04(4)	?	539.15(5)	?	668.14(7)	$C_{54}H_{20}^+$
206.07(2)	$[^{13}C]C_{15}H_9O^+$	316.11(3)	$[^{13}C]C_{25}H_{15}^+$	422.09(4)	$C_{30}H_{14}O_3^+$	540.01(5)	?	669.15(7)	$[^{13}C]C_{54}H_{20}^+$
206.11(2)	?	317.11(3)	?	423.10(4)	$[^{13}C]C_{30}H_{14}O_3^+$	541.03(5)	?	670.15(7)	$C_{54}H_{22}^+$
207.09(2)	$C_{15}H_{11}O^+$	318.00(3)	?	424.11(4)	$C_{34}H_{16}^+$	542.05(5)	?	671.16(7)	$[^{13}C]C_{54}H_{22}^+$
208.09(2)	$[^{13}C]C_{15}H_{11}O^+$	318.1(3)	$C_{24}H_{14}O^+$	425.13(4)	$[^{13}C]C_{34}H_{16}^+$	543.03(5)	?	672.17(7)	$C_{54}H_{24}^+$
209.03(2)	?	319.01(3)	?	426.14(4)	$C_{34}H_{18}^+$	544.08(5)	$C_{36}H_{16}O_6^+$	673.03(7)	?
210.01(2)	?	319.11(3)	$[^{13}C]C_{24}H_{14}O^+$	427.13(4)	$[^{13}C]C_{34}H_{18}^+$	545.11(5)	$[^{13}C]C_{36}H_{16}O_6^+$	673.17(7)	$[^{13}C]C_{54}H_{24}^+$
213.07(2)	$C_{17}H_9^+$	320.01(3)	?	428.13(4)	?	546.12(5)	$C_{44}H_{18}^+$	675.04(7)	?
214.07(2)	$[^{13}C]C_{17}H_9^+$	321.02(3)	?	429.01(4)	?	547.13(5)	$[^{13}C]C_{44}H_{18}^+$	677.07(7)	?
215.09(2)	$C_{17}H_{11}^+$	322.03(3)	?	429.13(4)	?	548.14(5)	$C_{44}H_{20}^+$	679.12(7)	$C_{55}H_{19}^+$
216.09(2)	$[^{13}C]C_{17}H_{11}^+$	323.04(3)	?	431.01(4)	?	549.13(5)	$[^{13}C]C_{44}H_{20}^+$	680.13(7)	$[^{13}C]C_{55}H_{19}^+$
217.10(2)	?	324.08(3)	$C_{26}H_{12}^+$	432.03(4)	?	550.14(6)	$C_{44}H_{22}^+$	681.14(7)	$C_{55}H_{21}^+$
218.08(2)	$C_{16}H_{10}O^+$	325.10(3)	$[^{13}C]C_{26}H_{12}^+$	433.05(4)	$C_{27}H_{13}O_6^+$	551.02(6)	?	682.16(7)	$[^{13}C]C_{55}H_{21}^+$
219.08(2)	$[^{13}C]C_{16}H_{10}O^+$	326.10(3)	$C_{26}H_{14}^+$	434.06(4)	$[^{13}C]C_{27}H_{13}O_6^+$	551.15(6)	$[^{13}C]C_{44}H_{22}^+$	683.16(7)	$C_{55}H_{23}^+$
220.09(2)	$C_{16}H_{12}O^+$	327.11(3)	$[^{13}C]C_{26}H_{14}^+$	435.11(4)	$C_{35}H_{15}^+$	553.02(6)	?	684.14(7)	$[^{13}C]C_{55}H_{23}^+$
221.00(2)	?	328.12(3)	?	436.11(4)	$[^{13}C]C_{35}H_{15}^+$	554.04(6)	?	685.16(7)	?
221.10(2)	$[^{13}C]C_{16}H_{12}O^+$	329.10(3)	$C_{25}H_{13}O^+$	437.13(4)	$C_{35}H_{17}^+$	555.06(6)	?	686.03(7)	?
222.01(2)	?	330.10(3)	$[^{13}C]C_{25}H_{13}O^+$	438.13(4)	$[^{13}C]C_{35}H_{17}^+$	556.06(6)	?	688.04(7)	?
222.04(2)	?	331.00(3)	?	439.14(4)	$C_{35}H_{19}^+$	557.11(6)	$C_{45}H_{17}^+$	690.09(7)	?
223.02(2)	?	332.01(3)	?	440.00(4)	?	558.13(6)	$[^{13}C]C_{45}H_{17}^+$	691.09(7)	?
224.02(2)	?	333.02(3)	?	440.14(4)	$[^{13}C]C_{35}H_{19}^+$	559.14(6)	$C_{45}H_{19}^+$	692.13(7)	$C_{56}H_{20}^+$
224.05(2)	$C_{14}H_8O_3^+$	334.04(3)	?	441.14(4)	?	560.13(6)	$[^{13}C]C_{45}H_{19}^+$	693.13(7)	$[^{13}C]C_{56}H_{20}^+$
225.03(2)	?	335.06(3)	$C_{23}H_{11}O_3^+$	442.01(4)	?	561.14(6)	$C_{45}H_{21}^+$	694.14(7)	$C_{56}H_{22}^+$
225.05(2)	$[^{13}C]C_{14}H_8O_3^+$	336.07(3)	$[^{13}C]C_{23}H_{11}O_3^+$	443.02(4)	?	562.15(6)	$[^{13}C]C_{45}H_{21}^+$	695.14(7)	$[^{13}C]C_{56}H_{22}^+$
226.08(2)	$C_{18}H_{10}^+$	337.10(3)	$C_{27}H_{13}^+$	444.03(4)	?	563.15(6)	?	696.15(7)	$C_{56}H_{24}^+$
227.08(2)	$[^{13}C]C_{18}H_{10}^+$	338.10(3)	$[^{13}C]C_{27}H_{13}^+$	445.04(4)	?	564.03(6)	?	697.15(7)	$[^{13}C]C_{56}H_{24}^+$
228.09(2)	$C_{18}H_{12}^+$	339.11(3)	$C_{27}H_{15}^+$	446.08(4)	$C_{32}H_{14}O_3^+$	566.04(6)	?	699.04(7)	?
229.10(2)	$[^{13}C]C_{18}H_{12}^+$	340.12(3)	$[^{13}C]C_{27}H_{15}^+$	447.10(4)	$[^{13}C]C_{32}H_{14}O_3^+$	567.04(6)	?	701.09(7)	?
230.11(2)	?	341.12(3)	$C_{27}H_{17}^+$	448.11(4)	$C_{36}H_{16}^+$	568.07(6)	$C_{38}H_{16}O_6^+$	703.11(7)	$C_{57}H_{19}^+$
231.08(2)	$C_{17}H_{11}O^+$	341.98(3)	?	449.12(4)	$[^{13}C]C_{36}H_{16}^+$	569.09(6)	$[^{13}C]C_{38}H_{16}O_6^+$	704.13(7)	$[^{13}C]C_{57}H_{19}^+$
232.09(2)	$[^{13}C]C_{17}H_{11}O^+$	342.12(3)	$[^{13}C]C_{27}H_{17}^+$	450.13(5)	$C_{36}H_{18}^+$	570.13(6)	$C_{46}H_{18}^+$	705.14(7)	$C_{57}H_{21}^+$
233.00(2)	?	343.11(3)	?	451.13(5)	$[^{13}C]C_{36}H_{18}^+$	571.13(6)	$[^{13}C]C_{46}H_{18}^+$	706.15(7)	$[^{13}C]C_{57}H_{21}^+$
233.10(2)	$C_{17}H_{13}O^+$	344.01(3)	?	452.14(5)	?	572.14(6)	$C_{46}H_{20}^+$	707.15(7)	$C_{57}H_{23}^+$
234.01(2)	?	345.02(3)	?	453.01(5)	?	573.13(6)	$[^{13}C]C_{46}H_{20}^+$	708.15(7)	$[^{13}C]C_{57}H_{23}^+$
235.01(2)	?	346.03(3)	?	453.13(5)	?	574.14(6)	?	709.17(7)	?
235.04(2)	$C_{15}H_7O_3^+$	347.04(3)	?	455.02(5)	?	575.02(6)	?	714.07(7)	?
236.04(2)	$[^{13}C]C_{15}H_7O_3^+$	348.07(3)	$C_{24}H_{12}O_3^+$	456.02(5)	?	575.14(6)	?	716.14(7)	$C_{58}H_{20}^+$
237.06(2)	$C_{19}H_9^+$	349.08(3)	$[^{13}C]C_{24}H_{12}O_3^+$	457.04(5)	?	577.03(6)	?	717.14(7)	$[^{13}C]C_{58}H_{20}^+$
238.07(2)	$[^{13}C]C_{19}H_9^+$	350.10(4)	$C_{28}H_{14}^+$	458.04(5)	?	579.04(6)	?	718.15(7)	$C_{58}H_{22}^+$
239.09(2)	$C_{19}H_{11}^+$	351.11(4)	$[^{13}C]C_{28}H_{14}^+$	459.11(5)	$C_{33}H_{15}O_3^+$	580.07(6)	?	719.14(7)	$[^{13}C]C_{58}H_{22}^+$

240.09(2)	$[^{13}C]C_{19}H_{12}^+$	352.11(4)	$C_{28}H_{16}^+$	460.11(5)	$C_{33}H_{16}O_3^+$	581.11(6)	$C_{47}H_{17}^+$	720.15(7)	?
241.10(2)	?	353.12(4)	$[^{13}C]C_{28}H_{16}^+$	461.13(5)	$C_{37}H_{17}^+$	582.12(6)	$[^{13}C]C_{47}H_{17}^+$	725.05(7)	?
242.09(2)	$C_{18}H_{10}O^+$	354.13(4)	$C_{28}H_{18}^+$	462.13(5)	$[^{13}C]C_{37}H_{17}^+$	583.14(6)	$C_{47}H_{19}^+$	727.10(7)	?
243.09(2)	$C_{18}H_{11}O^+$	355.00(4)	?	463.13(5)	$C_{37}H_{19}^+$	584.14(6)	$[^{13}C]C_{47}H_{19}^+$	729.14(7)	$C_{59}H_{21}^+$
244.09(2)	$C_{18}H_{12}O^+$	355.14(4)	$[^{13}C]C_{28}H_{18}^+$	464.01(5)	?	585.15(6)	$C_{47}H_{21}^+$	730.14(7)	$[^{13}C]C_{59}H_{21}^+$
245.10(2)	$[^{13}C]C_{18}H_{12}O^+$	356.01(4)	?	464.14(5)	$[^{13}C]C_{37}H_{19}^+$	586.15(6)	$[^{13}C]C_{47}H_{21}^+$	731.15(7)	$C_{59}H_{23}^+$
246.01(2)	?	356.14(4)	?	465.13(5)	?	587.16(6)	?	732.15(7)	$[^{13}C]C_{59}H_{23}^+$
247.02(2)	?	357.01(4)	?	466.02(5)	?	588.01(6)	?	733.17(7)	?
248.02(2)	?	358.03(4)	?	468.02(5)	?	588.14(6)	?	738.06(7)	?
248.05(2)	$C_{16}H_8O_3^+$	359.02(4)	?	469.04(5)	?	590.04(6)	?	740.13(7)	$C_{60}H_{20}^+$
249.05(2)	$[^{13}C]C_{16}H_8O_3^+$	360.06(4)	?	470.05(5)	?	591.05(6)	?	741.13(7)	$[^{13}C]C_{60}H_{20}^+$
250.07(3)	$C_{20}H_{10}^+$	361.09(4)	$C_{25}H_{13}O_3^+$	471.08(5)	?	592.06(6)	?	742.16(7)	$C_{60}H_{22}^+$
251.08(3)	$[^{13}C]C_{20}H_{10}^+$	362.10(4)	$[^{13}C]C_{25}H_{13}O_3^+$	472.11(5)	$C_{38}H_{16}^+$	593.08(6)	?	743.14(7)	$[^{13}C]C_{60}H_{22}^+$
252.09(3)	$C_{20}H_{12}^+$	363.11(4)	$C_{29}H_{15}^+$	473.13(5)	$[^{13}C]C_{38}H_{16}^+$	594.12(6)	$C_{48}H_{18}^+$	744.15(7)	?
253.1(3)	$[^{13}C]C_{20}H_{12}^+$	364.12(4)	$[^{13}C]C_{29}H_{15}^+$	474.12(5)	$C_{38}H_{18}^+$	595.13(6)	$[^{13}C]C_{48}H_{18}^+$	751.09(8)	?
254.10(3)	?	365.12(4)	$C_{30}H_{17}^+$	475.13(5)	$[^{13}C]C_{38}H_{18}^+$	596.14(6)	$C_{48}H_{20}^+$	753.13(8)	$C_{61}H_{21}^+$
255.08(3)	$C_{19}H_{11}O^+$	366.13(4)	$[^{13}C]C_{29}H_{17}^+$	476.13(5)	$C_{38}H_{20}^+$	597.15(6)	$[^{13}C]C_{48}H_{20}^+$	754.15(8)	$[^{13}C]C_{61}H_{21}^+$
256.08(3)	$C_{19}H_{12}O^+$	367.13(4)	?	476.99(5)	?	598.15(6)	$C_{48}H_{22}^+$	755.14(8)	$C_{61}H_{23}^+$
256.99(3)	?	368.01(4)	?	477.14(5)	$[^{13}C]C_{38}H_{20}^+$	599.15(6)	$[^{13}C]C_{48}H_{22}^+$	756.15(8)	$[^{13}C]C_{61}H_{23}^+$
257.09(3)	$C_{19}H_{13}O^+$	368.14(4)	?	479.02(5)	?	600.15(6)	?	757.16(8)	?
258.00(3)	?	369.02(4)	?	480.02(5)	?	601.02(6)	?	764.12(8)	?
259.00(3)	?	369.12(4)	?	481.04(5)	?	603.04(6)	?	766.14(8)	$C_{62}H_{22}^+$
259.04(3)	$C_{17}H_7O_3^+$	370.03(4)	?	482.03(5)	?	604.05(6)	?	767.17(8)	$[^{13}C]C_{62}H_{22}^+$
260.03(3)	$[^{13}C]C_{17}H_7O_3^+$	371.02(4)	?	483.10(5)	$C_{35}H_{15}O_3^+$	605.10(6)	$C_{41}H_{17}O_6^+$	768.14(8)	$C_{62}H_{24}^+$
261.06(3)	$C_{17}H_9O_3^+$	371.14(4)	?	484.10(5)	$[^{13}C]C_{35}H_{15}O_3^+$	606.11(6)	$[^{13}C]C_{41}H_{17}O_6^+$	777.14(8)	$C_{63}H_{21}^+$
262.06(3)	$[^{13}C]C_{17}H_9O_3^+$	372.06(4)	$C_{26}H_{12}O_3^+$	485.12(5)	$C_{39}H_{17}^+$	607.13(6)	$C_{49}H_{19}^+$	779.15(8)	$C_{63}H_{23}^+$
263.08(3)	$C_{21}H_{11}^+$	373.07(4)	$[^{13}C]C_{26}H_{12}O_3^+$	486.13(5)	$[^{13}C]C_{39}H_{17}^+$	608.14(6)	$[^{13}C]C_{49}H_{19}^+$	781.16(8)	?
264.09(3)	$[^{13}C]C_{21}H_{11}^+$	374.10(4)	$C_{30}H_{14}^+$	487.13(5)	$C_{39}H_{19}^+$	609.14(6)	$C_{49}H_{21}^+$	788.11(8)	?
265.10(3)	$C_{21}H_{13}^+$	375.11(4)	$[^{13}C]C_{30}H_{14}^+$	488.13(5)	$[^{13}C]C_{39}H_{19}^+$	610.14(6)	$[^{13}C]C_{49}H_{21}^+$	790.12(8)	?
266.11(3)	$[^{13}C]C_{21}H_{13}^+$	376.11(4)	$C_{30}H_{16}^+$	489.14(5)	?	611.16(6)	$C_{49}H_{23}^+$	792.15(8)	$C_{64}H_{24}^+$
267.11(3)	?	377.12(4)	$[^{13}C]C_{30}H_{16}^+$	490.01(5)	?	612.02(6)	?	801.14(8)	?
268.10(3)	$C_{20}H_{12}O^+$	378.12(4)	$C_{30}H_{18}^+$	490.13(5)	?	612.16(6)	$[^{13}C]C_{49}H_{23}^+$	803.15(8)	?
269.10(3)	$[^{13}C]C_{20}H_{12}O^+$	378.99(4)	?	492.02(5)	?	614.04(6)	?		
270.01(3)	?	379.13(4)	$[^{13}C]C_{30}H_{18}^+$	493.03(5)	?	616.06(6)	?		
271.01(3)	?	380.12(4)	?	494.04(5)	?	617.06(6)	?		
272.05(3)	$C_{18}H_8O_3^+$	381.01(4)	?	495.05(5)	?	618.12(6)	$C_{50}H_{18}^+$		

Supplementary Table 1. Peak list used in the data reduction. The incertitude on the *m/z* is shown between brackets.

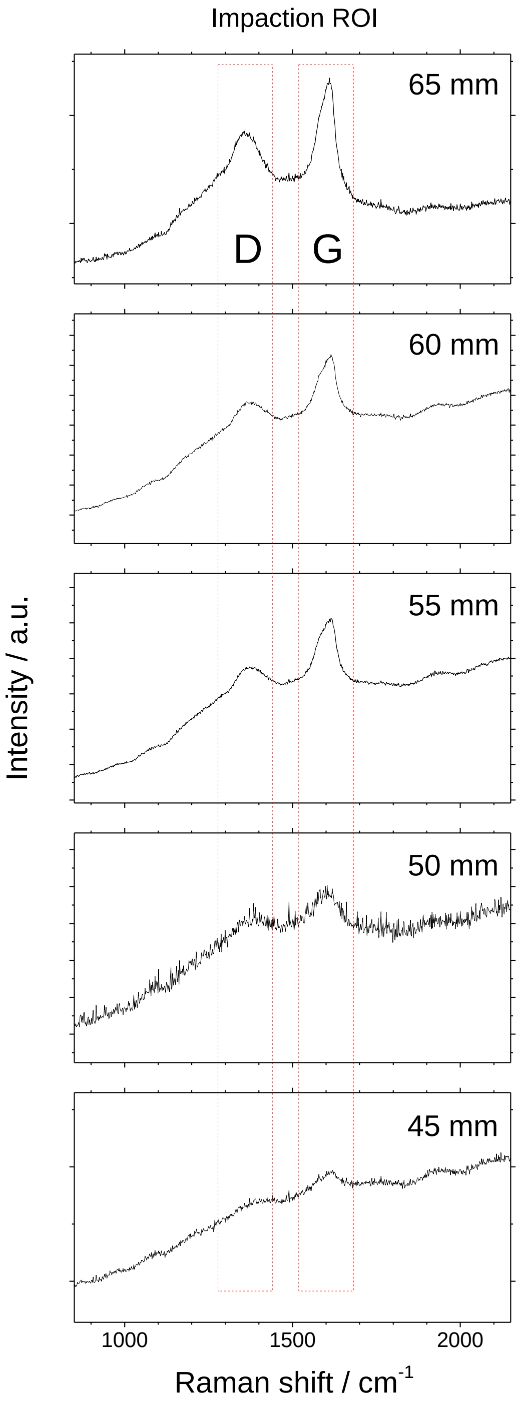

Supplementary Figure 4. Raman spectra of the impaction ROIs. Each row corresponds to one different sampling HAB.
